# Adverse drug events observed in patients with type 2 diabetes mellitus treated with 100 mg versus 300 mg canagliflozin: a systematic review and meta-analysis of published randomized controlled trials

**DOI:** 10.1186/s40360-017-0126-9

**Published:** 2017-04-16

**Authors:** Pravesh Kumar Bundhun, Girish Janoo, Feng Huang

**Affiliations:** 1 Institute of Cardiovascular Diseases, The First Affiliated Hospital of Guangxi Medical University, Nanning, Guangxi 530021 People’s Republic of China; 2 Guangxi Medical University, Nanning, Guangxi 530021 People’s Republic of China

**Keywords:** Canagliflozin, Adverse drug events, Type 2 diabetes mellitus, Oral hypoglycemic agents

## Abstract

**Background:**

Nowadays, canagliflozin monotherapy, or in combination with other oral hypoglycemic agents (OHAs), is often administered in patients who are treated for type 2 diabetes mellitus (T2DM). Therefore, we aimed to systematically compare the adverse drugs events (AEs) which were associated with 100 mg versus 300 mg canagliflozin respectively, using a large number of randomized patients with T2DM which were obtained from published trials.

**Methods:**

Randomized controlled trials (RCTs) comparing 100 mg versus 300 mg canagliflozin in patients who were treated for T2DM were searched from electronic databases. AEs reported during a follow up period ranging from 12 to 104 weeks were considered as the clinical endpoints in this analysis. We calculated odds ratios (OR) with 95% confidence intervals (CIs) and the analyses were carried out by RevMan 5 · 3 software.

**Results:**

Ten trials involving a total number of 5394 patients (2604 patients who were treated with 100 mg canagliflozin and 2790 patients who were treated with 300 mg canagliflozin) were included. The current results showed that serious AEs were not significantly higher in patients who were treated by 300 mg canagliflozin, with OR: 1.01, 95% CI: 0.79–1.29; *P* = 0.93. Also, a similar rate of death was observed in patients who were treated by either 100 or 300 mg canagliflozin with OR: 1.13, 95% CI: 0.43–2.94; *P* = 0.80. Urinary tract infections, postural dizziness and hypoglycemia were also similarly manifested, with OR: 0.93, 95% CI: 0.70–1.23; *P* = 0.61, OR: 1.51, 95% CI: 0.42–5.37; *P* = 0.53 and OR: 0.96, 95% CI: 0.81–1.13; *P* = 0.60 respectively. However, drug discontinuation due to AEs significantly favored 100 mg canagliflozin only during this unequal follow-up period with OR: 1.35, 95% CI: 1.06–1.72; *P* = 0.01, but it was not significantly different when trials with similar follow-up periods were analyzed.

**Conclusion:**

300 mg canagliflozin was not associated with significantly higher adverse events compared to 100 mg canagliflozin in those patients who were treated for T2DM. However, because this result was partly affected by other anti-diabetic medications which were included in the treatment regimen, further studies based on patients who were treated strictly on canagliflozin monotherapy should be recommended to completely solve this issue.

## Background

Canagliflozin is an inhibitor of subtype 2 sodium-glucose transport proteins (SGLT2) and it is often used to treat patients with type 2 diabetes mellitus (T2DM) [1]. This drug works by blocking glucose absorption in the kidneys, thereby eliminating glucose through urine [2, 3]. The mechanism of action of this drug renders it safe to be used and it results with less hypoglycemic events compared to other oral hypoglycemic agents (OHAs) such as sulfonyl urea, and even insulin. Canagliflozin can be administered alone (monotherapy), or in combination with other OHAs such as metformin, pioglitazone, sulfonylurea, and even insulin depending on the treatment regimen being followed. Several studies have compared canagliflozin with other OHAs showing the former to have more beneficial effects in these patients who were treated for T2DM [4].

Similar to other drugs, canagliflozin also has adverse drug effects (AEs). A systematic review and meta-analysis carried out by Yang et al confirmed common AEs such as genital tract infections, which are associated with the use of canagliflozin when it was compared to other OHAs [5]. Another meta-analysis showed genital mycotic infections and pollakiuria to be associated with the use of canagliflozin in comparison with another OHA [6]. However, the AEs associated with 100 mg versus 300 mg canagliflozin respectively, have seldom been studied. Therefore, the main objective of this analysis was to systematically compare the AEs which were associated with 100 mg versus 300 mg canagliflozin respectively, using a large number of randomized patients with T2DM which were obtained from published trials.

## Methods

### Data sources and search strategy

PubMed/Medline, EMBASE and the Cochrane databases were searched (from June to August 2016) for relevant English publications of randomized controlled trials (RCTs) comparing 100 mg with 300 mg canagliflozin in patients who were treated for T2DM. During this search process, the following terms were used:

- Canagliflozin and diabetes mellitus;
- Canagliflozin and type 2 diabetes mellitus;
- Sodium glucose co-transporter 2-inhibitor and diabetes mellitus;
- Canagliflozin 100 mg versus canagliflozin 300 mg;
- Canagliflozin and T2DM;
- SGLT2 and type 2 diabetes mellitus.

To further ensure that we did not miss any trial, the term ‘canagliflozin’ alone was also used, and all the trials obtained were cross checked once.

### Inclusion and exclusion criteria

Studies were included if:

- They were RCTs comparing 100 mg with 300 mg canagliflozin in patients who were treated for T2DM.
- They reported adverse drug events among their safety outcomes.
- They were published in English language.

Studies were excluded if:

- They were non-RCTs (meta-analyses, case studies or observational studies).
- They did not compare 100 mg with 300 mg canagliflozin in patients who were treated for T2DM.
- They compared 100 mg with 300 mg canagliflozin in patients who were treated for T2DM, but these trials only compared outcomes which were related to efficacy without comparing outcomes which were related to adverse drug events.
- They involved unpublished data.

### Endpoints, definitions and follow up periods

Adverse drug events which were reported in these trials (Table 1) were considered as the clinical endpoints in this analysis. The following AEs were reported:

**Table 1:** Reported adverse outcomes

Trials	Adverse events reported	Follow up (weeks)
Blonde 2016 [9]	Any AEs, AEs leading to drug discontinuation, hypoglycemia episodes	104
Forst 2014 [10]	Any AEs, AEs leading to discontinuation, serious AEs, deaths, UTI	52
Neal 2015 [11]	Any AEs, AEs causing discontinuation, serious AEs, deaths, UTI, documented and severe hypoglycemia	18
Gonzalez 2013 [12]	Any AEs, AEs leading to discontinuation, serious AEs, UTI, postural dizziness	52
Inagaki 2013 [13]	Hypoglycemia	12
Inagaki 2014	Discontinuation due to AEs	52
Stenlof 2013 [15]	Any AEs, AEs leading to discontinuation, serious AEs, death, UTI, postural dizziness	26
Wilding 2013 [16]	Any AEs, AEs leading to discontinuation, serious AEs, deaths, UTI, hypoglycemia	52
Yale 2013 [17]	Any AEs, AEs leading to discontinuation, serious AEs, deaths, UTI, postural dizziness	26
Rosenstock 2012 [18]	Any AEs, serious AEs, discontinuation due to AEs, UTI, hypoglycemia	12

*Abbreviations*: *AEs* adverse events, *UTI* urinary tract infections

- Any adverse events;
- Adverse events requiring drug discontinuation;
- Serious adverse events;
- Deaths;
- Urinary tract infections (UTIs);
- Postural dizziness;
- Hypoglycemia.

In this analysis, ‘any adverse event’ was reported in eight trials whereas adverse events leading to drug discontinuation were reported in nine trials. Seven trials reported serious adverse events and UTIs respectively. Death and hypoglycemia were reported in five trials each whereas postural dizziness was reported in three trials. One trial had a follow up period of 104 weeks, four trials had a follow up period of 52 weeks, and five other trials had a follow up period of less than or equal to 26 weeks (Table 1).

In this analysis, serious adverse events were defined as adverse events that were severe enough and required the assistance of another individual/medical staff or adverse events that resulted in seizures or/and loss of consciousness shortly following their occurrence.

UTIs were defined as infections which were related to the genitals, whether asymptomatic or with symptoms such as frequent urination, painful urination, any urethral discharge, or foul smelling of urinary organs.

Hypoglycemia was defined as a decrease in blood glucose level below 3.9 mmol/L (70 mg/dL).

### Data extraction and Quality assessment

Two authors (PKB and GJ) carefully assessed all the trials which were selected for this analysis. Information concerning the type of study which was reported, the total number of patients who were treated by 100 and 300 mg canagliflozin respectively, data concerning the baseline features, the reported adverse drug events and the corresponding follow up periods reported in the trials, were carefully extracted by these two authors. Any confusion or disagreement that followed was resolved by the third author (FH).

Bias risks which were observed in those eligible trials were assessed with reference to the components recommended by the Cochrane Collaboration [7] as shown in Table 2. The six components recommended by the Cochrane Handbook were assessed and a score of 0, 1 and 2 was given to a high, unclear and low risk of bias respectively. A minimum total score of 0 and a maximum total score of 12 was possible.

**Table 2:** Quality assessment of the trials according to the Cochrane Collaboration

Trials	A	B	C	D	E	F	Total score (n/12)
Blonde 2016 [9]	2	2	1	2	2	1	10
Forst 2014 [10]	2	2	1	1	2	1	9
Neal 2015 [11]	2	1	2	2	2	1	10
Gonzalez 2013 [12]	2	2	1	2	2	1	10
Inagaki 2013 [13]	2	2	1	1	2	1	9
Inagaki 2014	2	1	1	1	2	1	8
Stenlof 2013 [15]	2	1	2	2	2	1	10
Wilding 2013 [16]	2	1	2	2	2	1	10
Yale 2013 [17]	2	1	2	2	2	1	10
Rosenstock 2012 [18]	2	1	2	2	2	1	10

A: sequence generation; B: concealment of allocation; C: Blinding of patients; D: Blinding of caregivers; E: Blinding of outcome assessors; F: Follow-up and intention-to treat analysis

### Statistical analysis

The Preferred Reporting Items for Systematic Reviews and Meta*-*Analyses (PRISMA) guideline was followed for this analysis which involved only randomized trials [8]. Heterogeneity across the studies was calculated/assessed using the Cochrane Q-statistic test (*P* ≤ 0.05 was considered statistically significant) and the I^2^-statistic test (lower I^2^ value indicated a lower heterogeneity and higher I^2^ value represented an increasing heterogeneity).

In addition, a fixed (*I* ^2^ < 50%) or a random (*I* ^2^ > 50%) effects model was used based on the I^2^ values obtained.

Publication bias could visually be estimated by assessing funnel plots.

We calculated odds ratios (OR) with 95% confidence intervals (CIs) and the analyses were carried out by the RevMan 5 **·** 3 software.

### Ethics

Ethical approval was not required for this study.

## Results

### Search results

Six hundred and forty-five (645) articles were obtained from the searched databases. In the beginning, 529 articles were eliminated since they did not match with the scope of this analysis. One hundred and sixteen (116) full-text articles were assessed for eligibility. Further eliminations occurred based on the following criteria:

- Meta-analyses (7);
- Pooled analyses (8);
- They were different studies which involved the same trials (28);
- Duplicates (63).

**Fig. 1:**
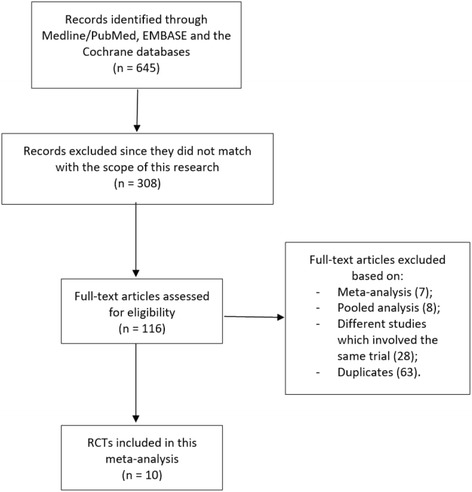
Flow diagram for the study selection

Finally, 10 trials [9–18] were considered eligible for this analysis (Fig. 1).

### General features of the included studies

A total number of 5394 patients were included (2604 patients were treated by 100 mg canagliflozin and 2790 patients were treated by 300 mg canagliflozin) as shown in Table 3.

**Table 3:** General features of the trials which were included in this analysis

Trials	Type of study	Patients enrollment period	No of patients using 100 mg CANA (n)	No of patients using 300 mg CANA (n)	Trial number
Blonde 2016 [9]	RCT	2009-2013	724	721	NCT00968812 + NCT01106651
Forst 2014 [10]	RCT	2010-2012	113	114	NCT01106690
Neal 2015 [11]	RCT	2009-2011	692	690	NCT01032629
Gonzalez 2013 [12]	RCT	2010-2012	368	367	NCT01106677
Inagaki 2013 [13]	RCT	2009-2010	74	75	NCT01022112
Inagaki 2014 [14]	RCT	2011-2012	127	253	NCT01387737
Stenlof 2013 [15]	RCT	2010-2012	195	197	NCT01081834
Wilding 2013 [16]	RCT	2010-2012	157	156	NCT01106625
Yale 2013 [17]	RCT	-	90	89	-
Rosenstock 2012 [18]	RCT	2008–2009	64	128	NCT00642278
Total no			2604	2790	

*Abbreviations*: *RCT* randomized controlled trials, *CANA* canagliflozin

The patients’ enrollment periods, the trial registration number, and the total number of patients treated with 100 and 300 mg canagliflozin respectively, have all been listed in Table 3.

According to Table 3, the enrollment periods of the patients varied between years 2008 and 2012.

### Baseline characteristics

The baseline features have been listed in Table 4.

**Table 4:** Baseline features of the participants

Trials	Mean age (y)	Males (%)	HbA1c (%)	Duration (y)	BMI (kg/m^2^)
	100/300 mg	100/300 mg	100/300 mg	100/300 mg	100/300 mg
Blonde 2016 [9]	56.4/55.8	52.2/49.7	7.8/7.8	6.5/6.7	31.0/31.2
Forst 2014 [10]	56.7/57.0	68.1/55.3	8.0/7.9	10.5/11.0	32.3/32.8
Neal 2015 [11]	62.0/63.0	67.0/65.0	8.3/8.3	16.4/16.3	33.0/33.3
Gonzalez 2013 [12]	55.5/55.3	47.3/45.0	7.9/7.9	6.7/7.1	32.4/31.4
Inagaki 2013 [13]	57.7/57.1	70.3/73.3	8.05/8.17	-	25.6/25.9
Stenlof 2013 [15]	55.1/55.3	41.5/45.2	8.1/8.0	4.5/4.3	31.3/31.7
Wilding 2013 [16]	57.4/56.1	48.4/55.8	8.1/8.1	9.0/9.4	33.3/33.2
Yale 2013 [17]	69.5/67.9	64.4/53.9	7.9/8.0	15.6/17.0	32.4/33.4
Rosenstock 2012 [18]	51.7/52.3	56.0/56.0	7.8/7.7	6.1/5.9	31.7/31.6

*Abbreviations*: *y* year, *HbA1c* glycosylated hemoglobin, *BMI* body mass index, Duration referred to duration of type 2 diabetes mellitus

100 and 300 mg were referred to canagliflozin dosage

According to Table 4, the participants had a mean age ranging from 51.7 to 69.5 years. Several studies reported a larger number of male patients compared to female patients. Glycosylated hemoglobin (HbA1c) ranged between 7.8 and 8.3%. Studies Neal [11] and Yale [17] involved patients with the longest duration period of T2DM as shown in Table 4.

Anti-diabetic drugs which were used by the patients were not same in all the trials. A few trials involved participants who used only canagliflozin monotherapy, whereas other trials involved participants who used canagliflozin in combination with either metformin, sulfonylurea, thiazolidinediones, or insulin as shown in Table 5.

**Table 5:** Other anti-diabetic drugs used by the patients apart from canagliflozin

Trials	Other anti-diabetic drugs used
Blonde 2016 [9]	Metformin
Forst 2014 [10]	Metformin and pioglitazone
Neal 2015 [11]	Metformin, sulfonyl urea and insulin
Gonzalez 2013 [12]	Metformin
Inagaki 2013 [13]	Only canagliflozin monotherapy
Inagaki 2014	Only canagliflozin monotherapy
Stenlof 2013 [15]	Only canagliflozin monotherapy
Wilding 2013 [16]	Metformin and sulfonyl urea
Yale 2013 [17]	Metformin, sulfonylurea, thiazolidinediones, or insulin
Rosenstock 2012 [18]	Metformin

### Adverse drug events analyzed

The main results of this analysis were summarized in Table 6.

**Table 6:** Results of this analysis

Outcomes reported	No of studies involved (n)	OR with 95% CI	P values	I^2^ (%)
Drug discontinuation	9	1.35 [1.06–1.72]	0.01	31
Serious AEs	7	1.01 [0.79–1.29]	0.93	0
Deaths	5	1.13 [0.43–2.94]	0.80	0
UTIs	7	0.93 [0.70–1.23]	0.61	0
P. dizziness	3	1.51 [0.42–5.37]	0.53	0
Hypoglycemia	5	0.96 [0.81–1.13]	0.60	0
Any AEs	8	1.04 [0.85–1.28]	0.72	52

*Abbreviations*: *AEs* adverse events, *UTIs* urinary tract infections, *P. dizziness* postural dizziness, *OR* odds ratios, *CI* confidence intervals

**Fig. 2:**
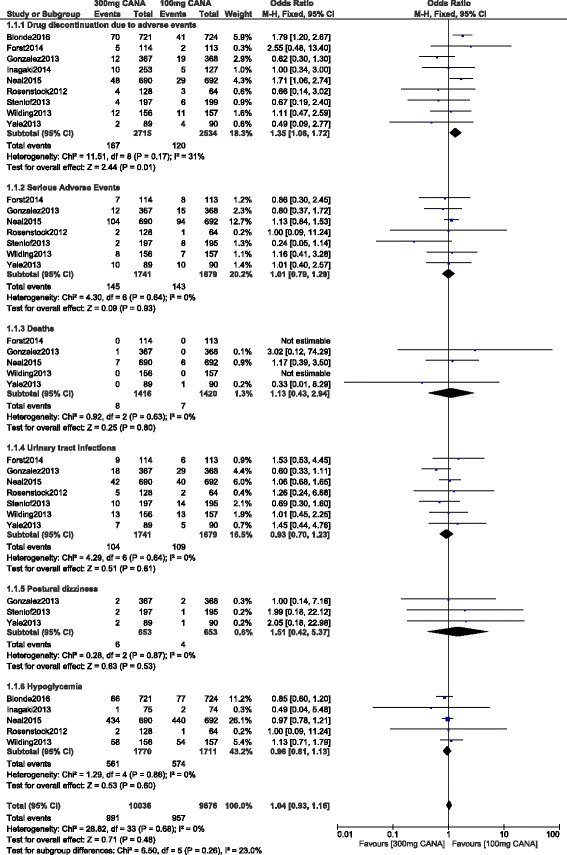
Adverse drug events associated with 100 and 300 mg canagliflozin (mean follow-up ranging from 12 to 104 weeks)

The current results showed that, during a follow up period ranging from 12 to 104 weeks, serious adverse events were not significantly higher in patients treated by 300 mg canagliflozin with OR: 1.01, 95% CI: 0.79–1.29; *P* = 0.93. Also, a similar rate of death has been observed in patients treated by either 100 or 300 mg canagliflozin with OR: 1.13, 95% CI: 0.43–2.94; *P* = 0.80. UTIs, postural dizziness and hypoglycemia were also similarly manifested with OR: 0.93, 95% CI: 0.70–1.23; *P* = 0.61, OR: 1.51, 95% CI: 0.42–5.37; *P* = 0.53 and OR: 0.96, 95% CI: 0.81–1.13; *P* = 0.60 respectively. However, drug discontinuation due to adverse events significantly favored 100 mg canagliflozin with OR: 1.35, 95% CI: 1.06–1.72; *P* = 0.01. These results have been illustrated in Fig. 2.

**Fig. 3:**
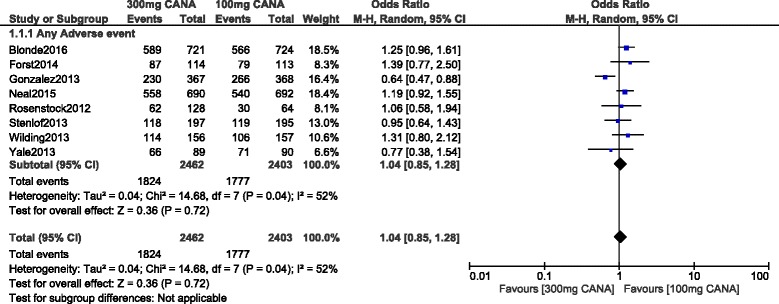
Any adverse drug events associated with 100 and 300 mg canagliflozin (mean follow-up ranging from 12 to 104 weeks)

Even if a moderate level of heterogeneity has been observed when analyzing ‘any type of adverse event’, no significant difference in events were observed whether with 100 or 300 mg canagliflozin with OR: 1.04, 95% CI: 0.85–1.28; *P* = 0.72. This result which was analyzed by a random effects model, has been represented in Fig. 3.

Because this main analysis involved a combination of trials with different follow-up periods, new analyses were further carried out with trials involving similar follow-up periods.

**Fig. 4:**
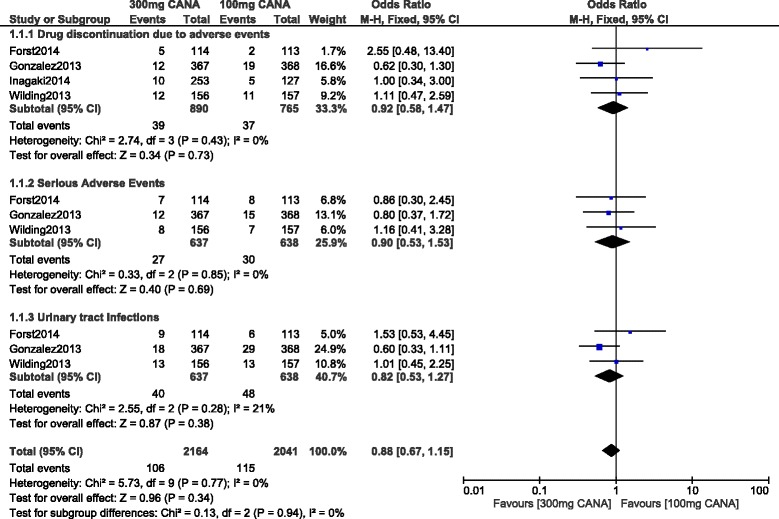
Adverse drug events associated with 100 and 300 mg canagliflozin (mean follow-up of 52 weeks)

**Fig. 5:**
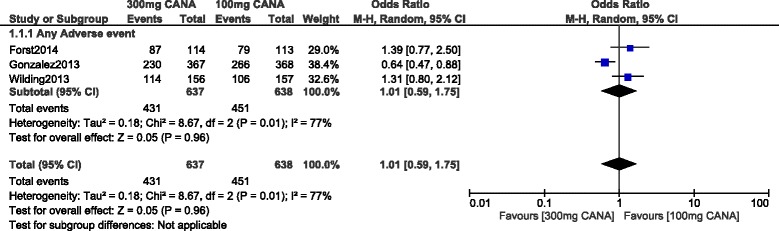
Any adverse drug events associated with 100 and 300 mg canagliflozin (mean follow-up of 52 weeks)

Four trials had a follow-up period of 52 weeks. When these four trials were analyzed separately from the main result, no significant difference was observed in serious adverse drug events and UTIs with OR: 0.90, 95% CI: 0.53–1.53; *P* = 0.69 and OR: 0.82, 95% CI: 0.53–1.27; *P* = 0.38 respectively. Drug discontinuation due to adverse events was also not significantly different with OR:0.92, 95% CI: 0.58–1.47; *P* = 0.73 as shown in Fig. 4. Any adverse event was also similarly manifested with OR: 1.01, 95% CI: 0.59–1.75; *P* = 0.96 (Fig. 5).

**Fig. 6:**
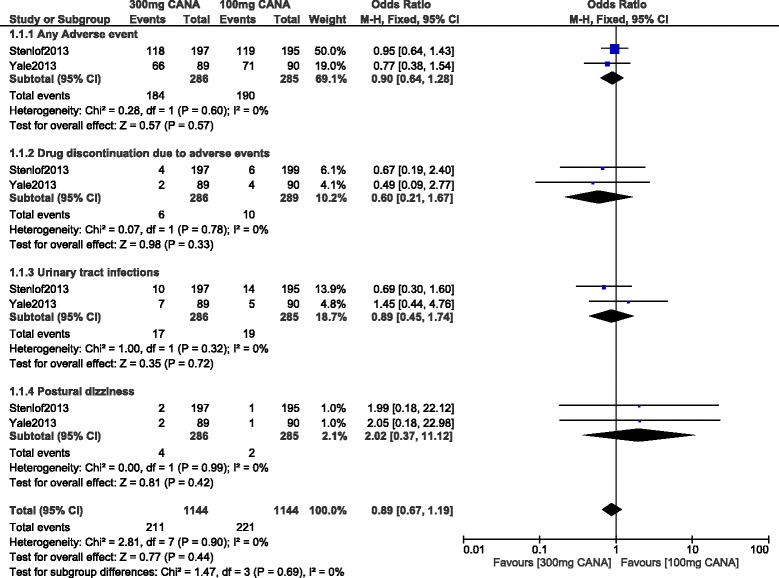
Adverse drug events associated with 100 and 300 mg canagliflozin (mean follow-up of 26 weeks)

When a follow-up period of 26 weeks was considered, any adverse event, drug discontinuation, UTIs and postural dizziness were still not significantly different with OR: 0.90, 95% CI: 0.64–1.28; *P* = 0.57, OR: 0.60, 95% CI: 0.21–1.67; *P* = 0.33, OR: 0.89, 95% CI: 0.45–1.74; *P* = 0.72 and OR: 2.02, 95% CI: 0.37–11.12; *P* = 0.42 respectively as shown in Fig. 6.

**Fig. 7:**
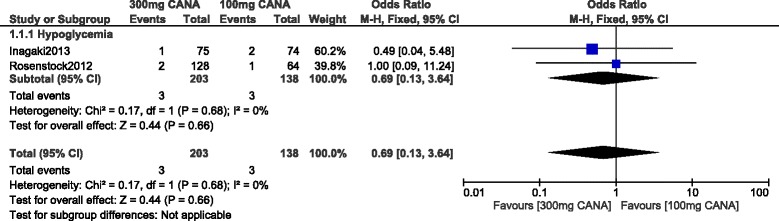
Hypoglycemia associated with 100 and 300 mg canagliflozin (mean follow-up of 12 weeks)

In addition, hypoglycemia was assessed during a follow-up period of 12 weeks. However, no significant difference was observed with OR: 0.69, 95% CI: 0.13–3.64; *P* = 0.66 (Fig. 7).

### Publication bias

**Fig. 8:**
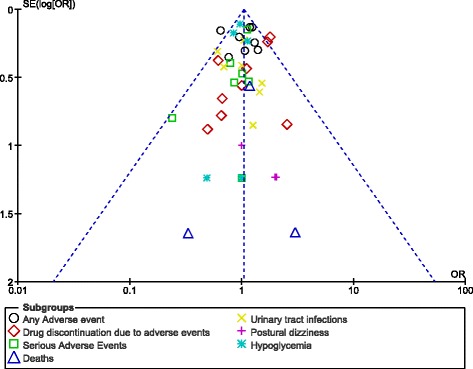
Funnel plot representing publication bias

**Fig. 9:**
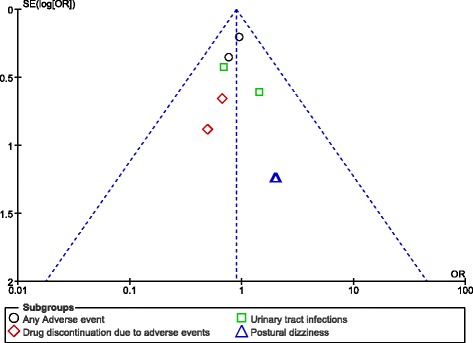
Funnel plot representing publication bias

After visually assessing the funnel plots, a low evidence of publication bias was observed across the trials involving several subgroups which assessed the adverse events associated with 100 mg versus 300 mg canagliflozin. The funnel plots representing publication bias have been illustrated in Figs. 8 and 9.

## Discussion

The current results showed no statistically significant differences in adverse drug events observed with 100 mg versus 300 mg canagliflozin in patients who were treated for T2DM. However, 300 mg dosage resulted in a significantly higher drug discontinuation only when a mean range of follow-up period (12 to 104 weeks) was considered but no significant difference in drug discontinuation was observed when trials with similar follow-up periods were compared.

Canagliflozin, which is a sodium-glucose transporter (SGLT-2) inhibitor, works by preventing the reabsorption of at least 90% of renal glucose, allowing the latter to be excreted in urine [2]. Risk of hypoglycemia is also minimal as compared to other anti-diabetic medications. This current result showed that neither 100 nor 300 mg canagliflozin caused any significant increase in hypoglycemia among those patients who were treated for T2DM. Canagliflozin’s mechanism of action also allows it to lower blood pressure in those patients with hypertension. In addition, canagliflozin also reduces postprandial glucose and insulin by delaying glucose absorption in the intestine [19]. In addition to the result of this analysis, no significant difference observed between 100 mg versus 300 mg canagliflozin could represent a potential benefit for similar patients in the future.

The pooled analysis of clinical studies comparing the efficacy and safety of canagliflozin in older patients suffering from T2DM showed canagliflozin to maintain a good glycemic control, to result in weight reduction, to improve blood pressure and it was well tolerated [20]. Another study using data pooled from four placebo-controlled phase 3 trials and two placebo-controlled sub-studies of a population of patients with inadequately controlled T2DM showed both dosages of canagliflozin (100 and 300 mg) to be well tolerated in Asians and in Black/African American [21]. Furthermore, Bode et al also showed canagliflozin to be well tolerated in patients with T2DM aged between 55 and 80 years over a follow up period of 2 years [22]. In addition of improving HbA1c, serious adverse events were not highly visible in patients who were treated with either 100 or 300 mg canagliflozin. A clinical review further discussed the safety of canagliflozin [23].

Nevertheless, the pooled analysis by Bailey et al involving two clinical trials showed that during a follow up period of 1 year, canagliflozin 100 mg was associated with a comparable attainment of composite quality endpoint whereas 300 mg canagliflozin was associated with superior composite quality endpoint attainment [24].

Despite so many benefits of canagliflozin, a recent study evaluating the safety of canagliflozin showed that the main adverse effect which was likely to be seen was a small increase in UTIs and fungal infections [25]. Scientific research has also shown the association of canagliflozin with an increase in vaginal colonization with candida species and it was also associated with vulvovaginal adverse effects in women with T2DM [26]. However, even if increased UTIs were observed with canagliflozin when compared to other OHAs, this current analysis showed that when two different dosages of the same drug were compared with each other, no significant difference was observed in UTIs. In addition, canagliflozin was also demonstrated to have potential adverse outcomes related to bones, thereby increasing the risk of fracture [27, 28].

Even if canagliflozin is associated with a small percentage of adverse drug events in these patients with T2DM, this current analysis showed that compared to a 100 mg dosage, a 300 mg dosage of canagliflozin was not associated with significantly higher adverse effects in these patients treated for T2DM, implying that both dosages are well tolerated in these patients [29].

It should be noted that canagliflozin in combination with other anti-diabetic drugs such as insulin, might improve glycemic control and reduce body weight in these patients with T2DM, as shown in the study published by Inagaki et al [30].

### Novelty

This study is new in the way that, it is among the first meta-analyses comparing the adverse drug events associated with 100 mg versus 300 mg canagliflozin in patients treated for T2DM. Several studies have previously compared other OHAs with canagliflozin, however, their main focus was not based on the adverse drug events which were associated with 100 mg versus 300 mg canagliflozin. Another novelty could be the fact that this analysis involved a high number of randomized patients which should be expected to provide robust results. Moreover, physicians from the department of endocrinology, and patients who are treated for T2DM might be very much concerned about the different dosages and adverse events associated with canagliflozin. Therefore, this hypothesis, hopefully, might be useful to a certain extent.

### Limitations

This analysis also has several limitations. First of all, due to a small population size when compared to other published meta-analyses outside the scope of this research, the current results might have been affected. Moreover, all the eligible trials which were used in this analysis did not report the same follow up periods which could have also affected the results. However, this limitation was partly solved when trials with similar follow up periods were compared. Moreover, to compare safety outcomes, randomized trials might not be the most recommended sources of evidence in comparison to observational studies and their subtypes. Therefore, this might also represent another limitation of this analysis. Another main limitation could be the fact that all the trials did not involve only patients who were treated with canagliflozin monotherapy. Majority of the patients were also treated by metformin and/or other anti-diabetic medications. It should be noted that this could have had a high impact on the results obtained.

## Conclusion

300 mg canagliflozin was not associated with significantly higher adverse events compared to 100 mg canagliflozin in those patients who were treated for T2DM. However, because this result was partly affected by other anti-diabetic medications which were included in the treatment regimen, further studies based on patients who were treated strictly on canagliflozin monotherapy should be recommended to completely solve this issue.

## Abbreviations

AEs: Adverse events
CI: Confidence intervals
OHAs: Oral hypoglycemic agents
OR: Odds ratios
RCTs: Randomized controlled trials
SGLT-2: Sodium-glucose transporter
T2DM: Type 2 diabetes mellitus
UTIs: Urinary tract infections

## References

[1] Sha S, Devineni D, Ghosh A (2011). Canagliflozin, a novel inhibitor of sodium glucose co-transporter 2, dose dependently reduces calculated renalthreshold for glucose excretion and increases urinary glucose excretion in healthy subjects. Diabetes Obes Metab.

[2] Bakris GL, Fonseca VA, Sharma K, Wright EM (2009). Renal sodium-glucose transport: role in diabetes mellitus and potential clinical implications. Kidney Int.

[3] Wright EM, Hirayama BA, Loo DF (2007). Active sugar transport in health and disease. J Intern Med.

[4] Leiter LA, Langslet G, Vijapurkar U, Davies MJ, Canovatchel W. Simultaneous Reduction in Both HbA1c and Body Weight with Canagliflozin Versus Glimepiride in Patients with Type 2 Diabetes on Metformin. Diabetes Ther. 2016;16. 10.1007/s13300-016-0163-1 PMC4900973 26984361

[5] Yang XP, Lai D, Zhong XY, Shen HP, Huang YL (2014). Efficacy and safety of canagliflozin in subjects with type 2 diabetes: systematic review and meta-analysis. Eur J Clin Pharmacol.

[6] Yang T, Lu M, Ma L, Zhou Y, Cui Y (2015). Efficacy and tolerability of canagliflozin as add-on to metformin in the treatment of type 2 diabetes mellitus: ameta-analysis. Eur J Clin Pharmacol.

[7] Higgins JPT, Altman DG. Assessing risk of bias in included studies. In: Higgins JPT, Green S, eds. Cochrane handbook for systematic reviews of interventions. Wiley; 2008. p. 187–241.

[8] Liberati A, Altman DG, Tetzlaff J (2009). The PRISMA statement for reporting systematic reviews and meta-analyses of studies that evaluate healthcareinterventions: explanation and elaboration. BMJ..

[9] Blonde L, Stenlöf K, Fung A, Xie J, Canovatchel W, Meininger G. Effects of canagliflozin on body weight and body composition in patients with type 2 diabetes over 104 weeks. Postgrad Med. 2016;128(4):371–80. 10.1080/00325481.2016.1169894 27002421

[10] Forst T, Guthrie R, Goldenberg R (2014). Efficacy and safety of canagliflozin over 52 weeks in patients with type 2 diabetes on background metformin and pioglitazone. Diabetes Obes Metab.

[11] Neal B, Perkovic V, de Zeeuw D (2015). Efficacy and safety of canagliflozin, an inhibitor of sodium-glucose cotransporter 2, when used in conjunction with insulin therapy in patients with type 2 diabetes. Diabetes Care.

[12] Lavalle-González FJ, Januszewicz A, Davidson J, Tong C, Qiu R, Canovatchel W, Meininger G (2013). Efficacy and safety of canagliflozin compared with placebo and sitagliptin in patients with type 2 diabetes onbackground metformin monotherapy: a randomised trial. Diabetologia.

[13] Inagaki N, Kondo K, Yoshinari T, Maruyama N, Susuta Y, Kuki H (2013). Efficacy and safety of canagliflozin in Japanese patients with type 2 diabetes: a randomized, double-blind, placebo-controlled, 12-week study. Diabetes Obes Metab.

[14] Inagaki N, Kondo K, Yoshinari T, Kuki H (2015). Efficacy and safety of canagliflozin alone or as add-on to other oral antihyperglycemic drugs in Japanesepatients with type 2 diabetes: A 52-week open-label study. J Diabetes Investig.

[15] Stenlöf K, Cefalu WT, Kim KA (2013). Efficacy and safety of canagliflozin monotherapy in subjects with type 2 diabetes mellitus inadequatelycontrolled with diet and exercise. Diabetes Obes Metab.

[16] Wilding JP, Charpentier G, Hollander P (2013). Efficacy and safety of canagliflozin in patients with type 2 diabetes mellitus inadequately controlled withmetformin and sulphonylurea: a randomised trial. Int J Clin Pract.

[17] Yale JF, Bakris G, Cariou B (2013). Efficacy and safety of canagliflozin in subjects with type 2 diabetes and chronic kidney disease. Diabetes Obes Metab.

[18] Rosenstock J, Aggarwal N, Polidori D, Canagliflozin DIA 2001 Study Group (2012). Dose-ranging effects of canagliflozin, a sodium-glucose cotransporter 2 inhibitor, as add-on to metformin in subjects with type 2 diabetes. Diabetes Care.

[19] Polidori D, Sha S, Mudaliar S, Ciaraldi TP, Ghosh A, Vaccaro N, Farrell K, Rothenberg P, Henry RR (2013). Canagliflozin lowers postprandial glucose and insulin by delaying intestinal glucose absorption in addition to increasing urinary glucose excretion: results of a randomized, placebo-controlled study. Diabetes Care.

[20] Sinclair A, Bode B, Harris S (2014). Efficacy and safety of canagliflozin compared with placebo in older patients with type 2 diabetes mellitus: a pooled analysis of clinical studies. BMC Endocr Disord..

[21] Gavin JR, Davies MJ, Davies M, Vijapurkar U, Alba M, Meininger G (2015). The efficacy and safety of canagliflozin across racial groups in patients with type 2 diabetes mellitus. Curr Med Res Opin.

[22] Bode B, Stenlöf K, Harris S (2015). Long-term efficacy and safety of canagliflozin over 104 weeks in patients aged 55-80 years with type 2 diabetes. Diabetes Obes Metab.

[23] Elmore LK, Baggett S, Kyle JA, Skelley JW (2014). A review of the efficacy and safety of canagliflozin in elderly patients with type 2 diabetes. Consult Pharm.

[24] Bailey RA, Vijapurkar U, Meininger G, Rupnow MF, Blonde L (2015). Diabetes-Related Composite Quality End Point Attainment: Canagliflozin Versus Sitagliptin Based on a Pooled Analysis of 2 Clinical Trials. Clin Ther.

[25] Boyle LD, Wilding JP (2014). A safety evaluation of canagliflozin : a first-in-class treatment for type 2 diabetes. Expert Opin Drug Saf.

[26] Nyirjesy P, Zhao Y, Ways K, Usiskin K (2012). Evaluation of vulvovaginal symptoms and Candida colonization in women with type 2 diabetes mellitustreated with canagliflozin, a sodium glucose co-transporter 2 inhibitor. Curr Med Res Opin.

[27] Blevins TC, Farooki A (2017). Bone effects of canagliflozin, a sodium glucose co-transporter 2 inhibitor, in patients with type 2 diabetes mellitus. Postgrad Med.

[28] Alba M, Xie J, Fung A, Desai M (2016). The effects of canagliflozin, a sodium glucose co-transporter 2 inhibitor, on mineral metabolism and bone in patients with type 2 diabetes mellitus. Curr Med Res Opin.

[29] Qiu R, Balis D, Xie J, Davies MJ, Desai M, Meininger G (2017). Longer-term safety and tolerability of canagliflozin in patients with type 2 diabetes: a pooled analysis. Curr Med Res Opin.

[30] Inagaki N, Harashima S, Maruyama N, Kawaguchi Y, Goda M, Iijima H (2016). Efficacy and safety of canagliflozin in combination with insulin: a double-blind, randomized, placebo-controlled study in Japanese patients with type 2 diabetes mellitus. Cardiovasc Diabetol..

